# Advanced Diabetic Nephropathy with Nephrotic Range Proteinuria: A Pilot Study of the Long-Term Efficacy of Subcutaneous ACTH Gel on Proteinuria, Progression of CKD, and Urinary Levels of VEGF and MCP-1

**DOI:** 10.1155/2013/489869

**Published:** 2013-09-12

**Authors:** J. A. Tumlin, C. M. Galphin, B. H. Rovin

**Affiliations:** ^1^Internal Medicine/Nephrology, University of Tennessee College Medicine, Chattanooga, TN 37403, USA; ^2^Southeast Renal Research Institute, 45 East Main Street, Chattanooga, TN 37408, USA; ^3^Renal Division, Ohio State University Wexner Medical Center, Columbus, OH 43210, USA

## Abstract

*Background and Objective.* Adrenocorticotropic hormone (ACTH) is able to reduce proteinuria in nondiabetic glomerulopathies through activation of melanocortin receptors (MCR) expressed in the podocyte. To determine the efficacy of ACTH, we conducted a randomized, open-label pilot trial of ACTH gel in patients with advanced diabetic nephropathy. *Study Design.* Twenty-three (23) patients with diabetic nephropathy were randomized to daily subcutaneous (SQ) injections of 16 or 32 units of ACTH gel for six months. *Outcome.* The primary endpoint was the percentage of patients achieving a complete remission (<300 mg/24 hours) within 6 months. Exploratory endpoints included the percentage of partial (50% reduction) remissions, changes in Cr, and urinary cytokine markers. *Results.* After 6 months of ACTH gel therapy, 8 of 14 (57%) patients achieved a complete (*n* = 1) or partial (*n* = 7) remission. In the low-dose ACTH gel group (16 units), urinary protein fell from 6709 + 953 to 2224 + 489 mg/24 hrs (*P* < 0.001). In contrast, 2 of 6 patients in the 32-unit group achieved partial remission, but aggregate proteinuria (5324 + 751 to 5154 + 853 mg/24 hours) did not change. Urinary VEGF increased from 388 to 1346 pg/mg urinary creatinine (*P* < 0.02) in the low-dose group but remained unchanged in the high-dose group. *Conclusion.* ACTH gel stabilizes renal function and reduces urinary protein for up to 6 months after treatment. The ClinTrials.gov identifier is NCT01028287.

## 1. Introduction

Diabetes mellitus is a significant and growing health problem in the United States and other developed countries. Despite increasing public awareness, improved glycemic control, and the wide spread use of renin-angiotensin antagonists [1], the high prevalence of diabetic nephropathy and other end-organ complications continues to be a major health concern [2]. Analysis of the United States Renal Data System (USRDS) database estimates that, by 2015, the prevalent dialysis population will exceed 700,000 with diabetes being the primary cause of end stage renal disease (ESRD) [3].

Recent studies suggest that injury to the podocyte and reduction in cell density are key to the development of diabetic nephropathy [4–7]. Multiple pathologic mechanisms contribute to podocyte dysfunction including hyperglycemia, advanced glycosylated end products, angiotensin II, and aldosterone induced oxidant injury [8–11]. Repeated injury from these pathways increases expression of transforming growth factor beta (TGF*β*) and inhibition of podocyte production of vascular endothelial growth factor (VEGF) activity [5, 12, 13]. Data from renal biopsies in patients with more advanced stages of diabetic nephropathy confirm that podocyte density falls with advancing disease and correlates with reduced VEGF expression [14–19]. Studies in cultured human podocytes suggest that acute depletion of VEGF with Bevacizumab contributes to podocyte loss through accelerated apoptosis and enhanced detachment from the basement membrane [20]. 

 Early studies as well as more current work find that adrenocorticotropin hormone (ACTH) can effectively reduce proteinuria in idiopathic membranous glomerulonephritis and other forms of glomerulopathy [21, 22]. While the mechanism is unknown, the observation that the melanocortin 1 receptors are expressed in glomerular podocytes [23] suggests that the protein lowering effects of ACTH may involve direct modification of podocyte function.

To investigate safety and efficacy of ACTH on proteinuria and progression of diabetic nephropathy, we conducted a randomized, open-label pilot study of two different doses of ACTH gel in patients with advanced diabetic nephropathy. Patients with refractory nephrotic syndrome despite concurrent ACE inhibitor therapy were consented and randomized to two different doses of ACTH for six months. Patients were monitored for changes in HgbA1c, change in serum Cr, and duration of remission. Additional objectives included investigating whether the effect of ACTH on proteinuria correlated with a change in urinary VEGF and MCP-1 levels. 

## 2. Patients and Methods

### 2.1. Study Population and Inclusion/Exclusion

The purpose of this pilot study was to determine the safety and efficacy of long-term (6 months) ACTH gel therapy in patients with advanced diabetic nephropathy. The protocol was reviewed and approved by a human investigations committee as having met all requirements of the Declaration of Helsinki. All of the patients enrolled in the trial gave a written informed consent prior to study participation and were informed that this research was supported by a Grant from the Questcor Corporation. 

Between April 2009 and June 2010, patients between the ages of 18 and 80 with refractory nephrotic syndrome secondary to Type I or Type II diabetes were screened for study participation. Refractory nephrotic syndrome from diabetes was defined as patients having a urine protein/Cr ratio over 3.0 while actively receiving ACE inhibitors and hyperglycemic therapy (i.e., insulin or oral hyperglycemic agents). The nephrotic syndrome was also considered refractory if the urine protein/Cr ratio was over 2.0 while receiving combination therapy with an ACE inhibitor and a second protein lowering agent (e.g., ARB, nondihydropyridine CCB, or spironolactone). 

Patients with estimated GFR less than 20 mLs/min by the MDRD formula were excluded from the study. Patients with known primary or secondary membranous GN, primary or secondary focal segmental glomerulosclerosis, or other nondiabetic forms of glomerulopathy were also excluded. To rule out the possibility of mixed glomerular lesions from diabetes and another primary glomerulopathy, six patients presenting with a urine protein/Cr ratio over 6.0 and rapidly declining renal function underwent diagnostic renal biopsies. All six patients were confirmed to have only glomerulopathies from advanced diabetes. Patients with positive serology tests for ANA, anti-ds-DNA, RPR, C-ANCA, P-ANCA, hepatitis B or C antibodies, or HIV were excluded. Patients were also excluded for HbA1c greater than 9%, dilated cardiomyopathies (EF < 40%), a history of diabetic ketoacidosis or hyperosmolar states during the preceding six months, or patients with an active or planned pregnancy during the study period. 

**Figure 1:**
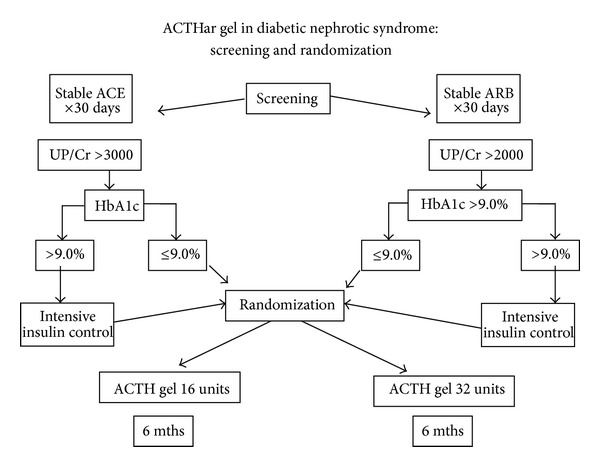


**Figure 2:**
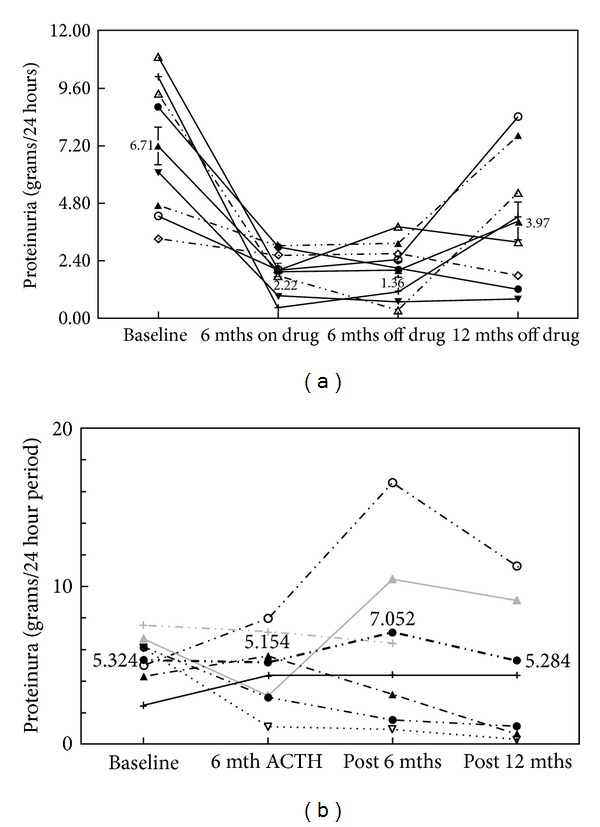
(a) 24-hour urinary protein (grams/24 hrs) is shown for each patient randomized to receive 16 units of ACTH gel SQ daily. Mean proteinuria decreased significantly after six months of therapy. Proteinuria tended to fall for 6 months after stopping ACTH therapy but did not reach statistical significance. (b) 24-hour urinary protein (grams/24 hrs) is shown for each patient randomized to receive 32 units of ACTH gel SQ daily. Mean proteinuria did not decrease after six months of therapy. Proteinuria tended to increase during the six months after stopping ACTH therapy but did not reach statistical significance. Two patients, including one with a late response, achieved a sustained partial remission.

During the screening period, all patients were required to have a stable blood pressure of less than 150/90 for four weeks while on a stable dose of an ACE inhibitor or combination therapy. A total of 49 diabetic patients (ages 31–74) with nephrotic syndrome were screened for study participation. Patients were screened using a spot protein/creatinine ratio, and proteinuria was then confirmed by two independent 24-hour urine collections. Patients that failed screening due to excess HgA1c were allowed to rescreen after modification of insulin regiment and achieving HbA1c below 9%, Figure 1. After giving a written informed consent, all patients meeting the inclusion exclusion criteria were randomized to receive daily subcutaneous injections of 16 (*n* = 8) or 32 (*n* = 6) units of ACTH gel for 6 months. Patients were seen in a followup on a monthly basis where adjustments to their insulin regimens or antihypertensive medications were made according to their blood pressure, fasting blood glucose, or HbA1c. The changes in urinary protein were monitored using monthly urine protein to creatinine ratios; see Figures 2(a) and 2(b). Additionally, blood pressure in all patients was targeted to be less than 140/90 mmHg. There were no changes in the dose of ACE, ARB, or other protein lowering agents during the 6-month study period. Dihydropteridine calcium channel blockers were preferentially used to maintain blood pressure control.

### 2.2. Control Population

To compare outcomes, patients matched for age, duration of diabetes, degree of proteinuria, prevalence of diabetic retinopathy, and CKD stage were used as case-cohort controls. All control patients were receiving stable ACE or ARB therapy and followed prospectively for 18 months. Data on patients' age, baseline creatinine, and level of proteinuria were recorded and compared to the two experimental groups.

#### 2.2.1. Primary/Secondary and Exploratory Endpoints

The primary endpoint of the study for the two experimental groups receiving ACTH was the percentage of patients achieving less than 300 mg protein per 24 hours after 6 months of therapy. Secondary endpoints included the percentage of patients achieving a partial response defined as a 50% reduction in urinary protein compared to the average of the two screening 24-hour urinary collections, the change in eGFR over 18 months, and the effect of ACTH on the urinary excretion of VEGF and MCP-1. Urinary protein was measured monthly in all patients during the treatment period and every 3 months for an additional 6 months after completion of the study in 13 of 14 patients and 12 months after completion in 11 patients. 

#### 2.2.2. Safety Endpoints

Patients enrolled in the study were monitored for changes in HbA1c on days 28, 56, and 84. The dose of insulin or oral hypoglycemic agents was modified to achieve an HbA1c value of 9% or less. Patients with blood glucose greater than 600 mg/dL despite the modification to insulin therapy underwent a reduction in ACTH gel dose or were withdrawn from the study. Patients could also be withdrawn from the study for glucose lability at the discretion of the principal investigator. Patients with edema refractory to diuretic therapy could either undergo a reduction of ACTH gel dose or be withdrawn from the study.

## 3. Urinary VEGF and MCP-1 Measurements

In an exploratory mechanism of action analysis, urinary levels of VEGF and monocyte chemoattractant peptide-1 (MCP-1) were measured at baseline and after 6 months of treatment. Using a Luminex assay and an enzyme-linked immunosorbant assay (ELISA), respectively, samples from each time point were measured in duplicate, and the amount of urinary VEGF or MCP-1 normalized to urinary creatinine. The methods for the uMCP-1 assay have been previously reported [24]. For uVEGF, urine samples were collected and stored at −80°C until being assayed. According to the manufacturer's protocol, urine was incubated with detecting antibody followed by stimulation of Luminex beads at 635 nanometers (nm) and fluorescence measurement at 532 nm. Fluorescence from all samples was then measured and VEGF concentration extrapolated from a standard curve. 

### 3.1. Statistical Analyses

Statistical analysis was conducted only on patients completing 6 months of ACTH gel therapy. Unless otherwise stated, data are expressed as mean ± standard error (S.E.M) for continuous variables or as a percentage for dichotomous variable. Statistical differences between baseline and posttreatment measurements of urinary protein, serum creatinine, and HgA1c were calculated using a Student's *t*-test and a two-sample paired analysis. For multiple comparisons, ANOVA followed by Bonferroni correction was used. Statistical calculations were conducted using SigmaPlot version 12.0. A *P* value of <0.05 was considered to be statistically significant. 

## 4. Results

Table 1 lists the demographic data of the 23 case-cohort controls and the 14 patients with type I (1 patient) or type II (13 patients) diabetes and refractory nephrotic syndrome who completed 6 months of ACTH therapy. The mean age was 58 ± 3, 53 ± 3, and 52.0 ± 2 years in the case-cohort controls, 16, and 32 unit groups, respectively. All patients had a greater than 10-year history of diabetes (17.1 ± 1, 17.1 ± 3, and 11.6 ± 2 years in the case-cohort controls, 16, and 32 unit groups, resp.). Baseline creatinine levels tended to be lower in the cohort controls compared to the two treatment groups, but this difference was not statistically significant (1.9 ± 0.2, 2.5 ± 0.5, and 2.7 ± 0.6 mg/dL), respectively (*P* = 0.156). While proteinuria was higher in the 16-unit compared to the 32-unit dose group (6709 ± 953 versus 5324 ± 428 mg/24 hours), this difference did not reach statistical significance (*P* = 0.716). The majority (57%) of patients in the study were receiving dual protein lowering therapy at the time of enrollment. A total of 6 of the 14 patients underwent diagnostic renal biopsies of which all were consistent with advanced diabetic nephropathy.

All of the patients had nephrotic range proteinuria at the time of enrollment with 7 (50%) patients excreting more than 6000 mg/d of protein (range 3315 to 10,914 mg/d). In Figure 2(a), the changes in protein excretion for each individual patient randomized to 16 units of ACTH gel are shown at baseline after 6 months of ACTH gel therapy and 6 months after completion of the study and stopping ACTH gel. The mean amount of proteinuria at baseline was 6709 ± 953 mg/ds, and this value fell to 2224 ± 489 mg/d after 6 months of ACTH gel treatment (*P* < 0.001). Interestingly, proteinuria in the 16-unit group continued to fall in 7 of 8 patients during the 6-month period after completion of the ACTH therapy. Six months after stopping ACTH gel injections, proteinuria tended to remain below baseline (1369 ± 380 mg/d, *P* = 0.180, compared to 6 months of active treatment). To determine the duration of the protein lowering effects of ACTH gel, urinary protein was measured in 11 of 14 patients 12 months after completion of the study. While the average proteinuria 12 months after completion of ACTH treatment increased to 3973 ± 400 mg/d; 3 of the 8 patients in the 16-unit group remained in partial remission. 

**Figure 3:**
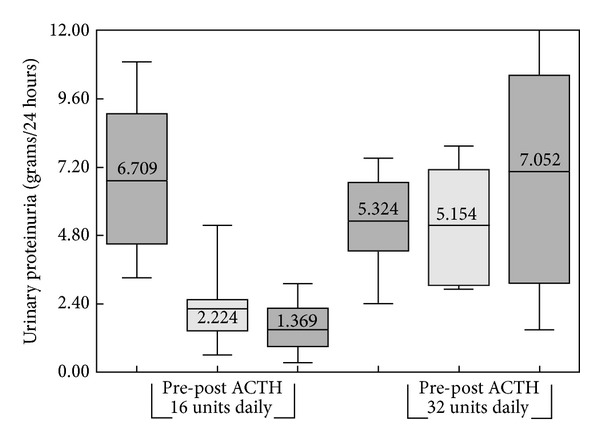
Whisker box plots of 24-hour urinary protein (grams/24 hrs) response for the 16- and 32-unit ACTH groups. A significant reduction in proteinuria is noted for the 16-unit dose at 6 months with a trend toward additional reduction in proteinuria 6 months after withdrawing ACTH. No effect is seen with the 32-unit ACTH dose.

**Figure 4:**
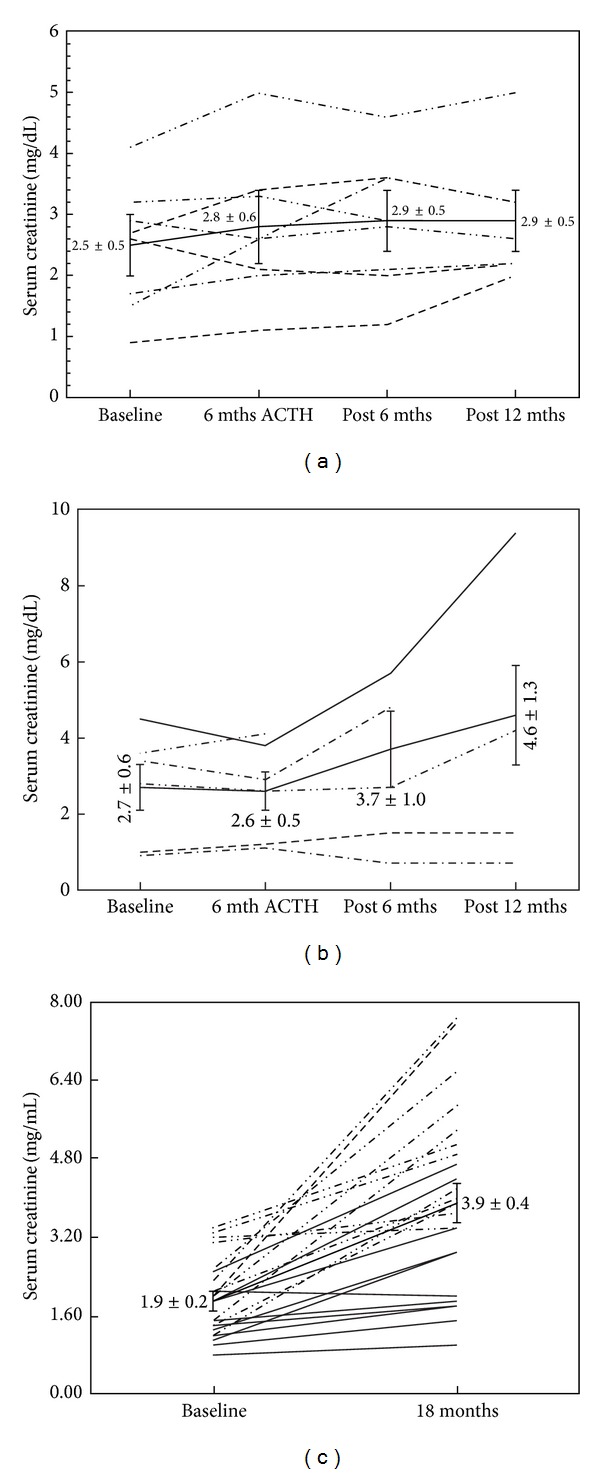
(a) The change in serum creatinine for patients randomized to the 16-unit dose from baseline to 12 months of completion of ACTH therapy. There was no significant change in average serum Cr. A single patient from this group developed ESRD within 17 months. (b) The change in serum creatinine for patients randomized to the 32-unit dose from baseline to 12 months of completion of ACTH therapy. There was a trend toward increased Cr at 12 months, but this difference did not reach statistical significance. Three of six patients in this group developed ESRD within 14.3 months. (c) The change in serum creatinine for age-matched diabetic controls not receiving ACTH therapy. Serum Cr significantly increased (*P* = 0.001) over 18 months. Of the 23 patients, 12 reached ESRD within 18 months.

Figure 2(b) shows the changes in proteinuria among those patients randomized to 32 units of ACTH gel. At baseline, the aggregate 24-hour proteinuria was 5324 ± 751 mg/d, which was not statistically different following 6 months of treatment or after six and twelve months of followup (5154 ± 853, 7052 ± 2278, and 5284 ± 2126 mg/d, resp.). Of the 6 patients randomized to 32 units, 2 showed a partial response at the end of the 6-month treatment. A single patient experienced a fall in proteinuria from 4261 mg/24 hrs at baseline to 619 mg/24 hrs 12 months after completion of the study.  Figure 3 illustrates the change in urinary protein for the 16- and 32-unit groups with 95% confidence intervals.  Figure 4(a) demonstrates the serial changes in serum Cr for patients enrolled in the 16-unit group at baseline, after 6 months of ACTH gel therapy, and at 6 and 12 months after the therapy. The mean serum Cr did not change significantly from baseline to 6 months of treatment or 6 and 12 months posttreatment (2.5 ± 0.5, 2.8 ± 0.6, 2.9 + 0.5 to 2.9 + 0.5 mg/dL) (*P* = 0.826). Similarly, eGFR at 18 months was not significantly different from baseline (34 ± 8.4 versus 21 ± 3.8 mLs/min, *P* = 0.175). For patients enrolled in the 32-unit group, serum Cr tended to rise (2.7 ± 0.6, 2.6 ± 0.5, 3.7 + 1.0 to 4.6 + 1.3 mg/dL), but this increase did not reach statistical significance (*P* = 0.402) (Figure 4(b)). In the 32-unit group, eGFR did not change over 18 months (39 ± 10.0 versus 30 ± 18 mLs/min, *P* = 0.617). Of the 14 patients completing 6 months of ACTH gel therapy, 4 progressed to ESRD with 1 patient and 3 patients from the 16- and 32-unit groups, respectively. In the control group (Figure 4(c)), no patient had achieved a complete or partial reduction in proteinuria during 18 months of observation. However, eGFR fell significantly (*P* = 0.0043) from 44 ± 4.6 mLs/min at baseline to 16 ± 4.3 mLs/min. Of the 23 controls, 12 patients required renal replacement therapy by 18 months. 

**Figure 5:**
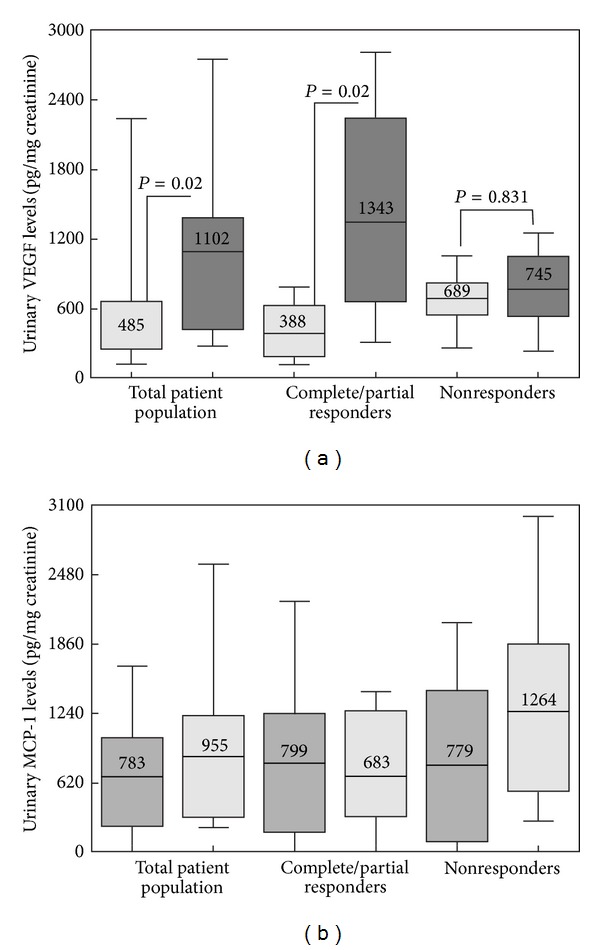
The change in urinary VEGF levels among the total group, complete and partial responders, and nonresponders. Urinary VEGF was significantly increased in patients demonstrating complete or partial reduction in urinary protein. (b) The change in urinary MCP-1 levels among the total group, complete and partial responders, and nonresponders. Urinary MCP-1 was unchanged by ACTH therapy.

We measured uVEGF and uMCP-1 in ACTH gel responsive and nonresponsive patients. All data were reported in picograms (pg) of VEGF or MCP-1 per milligram of urinary creatinine (Figure 5(a)). In the total population, urinary VEGF levels rose significantly from 485 ± 90 to 1102 ± 286 pg/mg Cr (*P* < 0.02). Among the patients achieving a complete or partial response, urinary VEGF levels rose from 388 ± 81 to 1346 ± 299 pg/mg Cr (*P* < 0.02) but remained unchanged among nonresponsive patients (689 ± 80 to 749 ± 127 pg/mg Cr, *P* = 0.564). In contrast, uMCP-1 levels were unchanged from baseline after 6 months of ACTH gel treatment in both responsive and nonresponsive patients (see Figure 5(b)).

## 5. Safety and Adverse Events

During 6 months of ACTH gel therapy, mean HbA1c increased from 7.9 ± 0.4 to 8.0 ± 0.5% (16 unit dose) and from 8.0 ± 0.5 to 8.7 ± 0.4% (32 unit dose). Of the 23 randomized patients, 14 completed 6 months of ACTH gel therapy. Four patients (2 in the 16 and 2 in the 32-unit groups) developed refractory edema. Three of these patients responded to modification of diuretics, and two patients chose to withdraw from the study. Two patients in the 32-unit group and one in the 16-unit group developed significant hyperglycemia. The dose of ACTH gel was reduced in two patients. This led to improved glycemic control in one patient; two patients chose to withdraw from the study. Two patients withdrew because of the need for additional injections and one because of severe insomnia. Three patients withdrew because of scheduling conflicts and the need for monthly followup. A single patient experienced hypotension following withdrawal from ACTH therapy that was treated successfully with oral steroid hormones and a slower taper of the drug. 

## 6. Discussion

We have conducted a randomized, prospective pilot trial of two doses of ACTH gel in patients with refractory nephrotic syndrome secondary to diabetic nephropathy to investigate the safety and efficacy of ACTH gel in reducing proteinuria and stabilizing renal function. We found that ACTH induced a complete or partial remission in 57% of patients. We further demonstrated that ACTH appears to have favorable long-term effects with sustained reductions in proteinuria and stable renal function up to 12 months following drug therapy. Investigating potential mechanisms of ACTH action, we found that ACTH increased urinary VEGF excretion among those patients who achieved a reduction in proteinuria. 

The widespread use of antagonists of the renin angiotensin system has significantly impacted the progression of diabetic nephropathy in both the early and late stages of the disease [1]. While novel agonists of antioxidant pathways and other approaches show promise in diabetic nephropathy [25], the development of overt proteinuria typically leads to progressive renal disease and the eventual need for renal replacement therapy [2]. Diabetic nephropathy is characterized by expansion of mesangial matrix, thickening of the basement membranes, and development of sclerosis in areas of reduced podocyte density [10, 26]. The progressive loss of podocyte function is increasingly being recognized as a central pathogenic event in diabetic nephropathy. White et al. demonstrated that urinary albumin excretion rates were inversely proportional to the number of glomerular podocytes [26]. Reduction in podocyte density has a significant clinical impact as noted by Meyer et al. who reported that the number of podocytes per glomerulus was the strongest predictor of progressive diabetic nephropathy [5, 10, 15]. The mechanisms contributing to progressive loss of podocytes include hyperglycemia, angiotensin II and aldosterone induced oxidant injury, and increased production of TGF*β* [9–11]. Recent work in cultured podocytes found a biphasic effect of TGF*β* on VEGF production and rates of podocyte apoptosis [12, 13, 27]. It is thought that repeated injury from these pathways leads to progressive loss of podocyte number and VEGF production.

Recent clinical studies have examined the utility of adrenocorticotropic hormone (ACTH) in the treatment of the nephrotic syndrome from a variety of different glomerulopathies [22]. ACTH is a 39-aminoacid peptide that is a member of the melanocortin family of proteins. ACTH signals through five widely distributed melanocortin receptors that have recently been demonstrated in glomerular podocytes [23]. The observation that melanocortin receptors are present in podocytes raises the possibility that ACTH alters glomerular permeability by modifying podocyte function. Previous studies have found that ACTH therapy can significantly reduce proteinuria in immune- and nonimmune-mediated glomerulopathies [21, 28]. Our observation that ACTH gel can reduce proteinuria in patients with advanced diabetic nephropathy suggests that the protein lowering effects of ACTH may be independent of its anti-inflammatory properties and possibly involve stabilization of podocyte function through activation of MCR-1 receptors [24, 28]. ACTH gel is a porcine pituitary extract containing full-length ACTH_(1-39)_ and perhaps small amounts of other proopiomelanocortin derived peptides [29]. It is unclear whether the effects of ACTH gel are due primarily to intact ACTH or to the simultaneous activation of multiple melanocortin receptors by other proopiomelanocortin peptides [22]. 

The majority of patients enrolled in our study had longstanding diabetes with a mean duration of over 10 years. The mean proteinuria at baseline was over 6000 mg/d indicating that our patients had severe diabetic nephropathy with a high likelihood of progression to end stage kidney disease. The primary endpoint of the study was the percentage of patients achieving a complete remission, defined as less than 300 mg protein per 24 hours. For patients completing 6 months of ACTH gel therapy, we found that urinary protein was reduced by 43%, with 57% of patients (8 of 14) achieving a complete or partial remission. Among those patients receiving the 16 unit dose, the effect of ACTH gel was more pronounced with a 67% reduction in protein and a 75% complete plus partial response rate. Like Berg and Arnadottir, we found that ACTH gel exhibited a prolonged effect on glomerular permeability with a total of 7 patients remaining in partial remission up to 12 months after withdrawal of the drug [28]. The protein lowering effect of the 32 unit dose was less pronounced. While two patients did achieve a partial response during the treatment period, none went into complete remission. These data suggest that the doses chosen for our study may have been on the high end of the dose-response curve and that lower doses of ACTH gel may have similar protein lowering effects with an even more favorable side-effect profile. The mechanisms for the reduced efficacy of the 32 unit dose are unknown. Potential explanations include the presence of oligopeptides or other degratory products of pituitary hormones that interfere with melanocortin receptor signaling at higher concentrations or that ACTH has a dose-dependent effect on podocyte function that is beneficial at lower doses but toxic at higher doses [12]. 

In the adrenal gland, ACTH maintains glandular capillary networks through its regulation of VEGF production [27, 30, 31]. Interestingly, recent clinical studies demonstrate that prolonged use of Bevacizumab and other VEGF antagonists can lead to proteinuria. In vitro studies in cultured podocytes showed that treatment with Bevacizumab induces podocyte apoptosis through activation of the p38/MAPK pathway [20]. While the role of VEGF in diabetic nephropathy is unclear, studies in patients with late-stage diabetic nephropathy find that glomerular expression of VEGF declines with advancing disease [16, 17]. In our study of patients with advanced diabetic nephropathy, we found that patients responsive to ACTH also demonstrated a significant rise in urinary VEGF. We did not, however, find a concomitant rise in uMCP-1, which was somewhat unexpected, given the putative involvement of MCP-1 in diabetic nephropathy and the known anti-inflammatory effects of ACTH [24]. More detailed mechanistic studies will need to be performed to determine the mechanism of ACTH on glomerular permeability

## 7. Summary

In summary, we conducted a prospective, open-label trial of ACTH gel in 14 patients with ACE inhibitor-resistant nephrotic syndrome secondary to advanced diabetic nephropathy. We showed that 6 months of therapy significantly reduces proteinuria and stabilizes renal function in over half the patients. Our preliminary results suggest a link between a reduction in proteinuria and rising levels of urinary VEGF. Larger studies with formal blinded controls will be needed to prove the efficacy of ACTH gel and to investigate its mechanism of action in diabetic nephropathy, but these data suggest that ACTH gel therapy may represent a novel approach for treatment of diabetic nephropathy.

## Figures and Tables

**Table 1:** 

Variable	Controls (*N* = 23)	ACTH 16 (*N* = 8)	ACTH 32 (*N* = 6)
Age (years)	53.8 ± 3	53.0 ± 3	52.0 ± 2
Male/female	11/23 (48% M)	4/8 (50% M)	4/6 (66% M)
Diabetes (yrs.)	17.7 ± 1	17.7 ± 3	11.6 ± 2
Retinopathy	47.8%	50.0%	66%
Serum Cr (mg/dL)	1.9 ± 0.2	2.2 ± 0.2	3.3 ± 0.3
Albumin (mg/dL)	3.1 ± 0.1	3.5 ± 0.1	3.0 ± 0.2
Proteinuria (gms/24 hrs)	5.6 ± 0.7	6.7 ± 9	5.3 ± 4
Dual Tx %	5/23 (22%)	5/8 (63%)	3/6 (50%)
Renal Bx	2/23 (8.6%)	2/8 (25%)	4/6 (66%)
